# Improving preparedness prior to reconstructive breast surgery via inclusion of 3D images during pre-operative counselling: a qualitative analysis

**DOI:** 10.1186/s12905-021-01463-6

**Published:** 2021-08-31

**Authors:** Alan D. McCrorie, Aislinn M. Begley, Jingwen J. Chen, Noleen K. McCorry, Glenda Paget, Stuart A. McIntosh

**Affiliations:** 1 The Patrick G Johnston Centre for Cancer Research, Queen’s University Belfast, 97 Lisburn Road, Belfast, BT9 7AE N.Ireland UK; 2 The Wellcome-Wolfson Institute for Experimental Medicine, Queen’s University Belfast, 97 Lisburn Road, Belfast, BT9 7BL N.Ireland UK; 3 Centre for Public Health, Queen’s University Belfast, Institute of Clinical Sciences, Block B, Royal Victoria Hospital, Belfast, BT12 6BA N.Ireland UK; 4 Belfast City Hospital, Belfast Health and Social Care Trust, 51 Lisburn Road, Belfast, BT9 7AB N.Ireland UK

## Abstract

**Background:**

A proportion of women undergoing mastectomy for breast cancer choose to undergo breast reconstruction. Evidence suggests that women’s preparedness for this surgery is low and that this may contribute to feelings of unmatched expectations and anxiety. There is substantial interest in decision-aids to remedy this. This study explores the incorporation of digitally rendered three-dimensional images into pre-operative counselling sessions as a means of enhancing patient preparedness.

**Methods:**

A database of three-dimensional images was produced showing both optimal and sub-optimal aesthetic outcome, matched to participant on the basis of type of surgical reconstruction, body habitus, and skin tone. Women undergoing mastectomy for breast cancer followed by immediate reconstruction were targeted for inclusion. Participants interacted with image software during pre-operative counselling sessions by viewing, rotating, and zooming in/out to gain a more in-depth appreciation of post-operative aesthetic outcome. Semi-structured face-to-face interviews followed thereafter. Interviews were audio-recorded, transcribed, coded, and themes identified.

**Results:**

Eight semi-structured interviews took place. The major emergent theme was ‘increased preparedness’ with subthemes including ‘expectation management’, ‘software interaction’, and ‘enhanced realism’. There were no prohibitively negative emotions after interacting with images. Women reported gaining ‘more of a perspective’ and feeling ‘more informed’ after viewing images. They also valued the enhanced interactivity and better appreciation of reconstructed breast symmetry that viewing three-dimensional images offered when compared to viewing two-dimensional photographs. Finally, women also commented that three-dimensional images were more realistic.

**Conclusions:**

Results suggest that incorporation of three-dimensional images into pre-operative counselling sessions prior to breast reconstruction, is a fairly simple yet effective method of enhancing patient preparedness prior to surgery. Women particularly valued the ability to use the software to generate a more realistic idea of what to expect after their operation. Future work should focus on better understanding any quantifiable benefit from incorporating three-dimensional images routinely into pre-operative decision-making.

**Supplementary Information:**

The online version contains supplementary material available at 10.1186/s12905-021-01463-6.

## Background

Approximately 20% of women undergoing mastectomy for breast cancer will choose to have immediate breast reconstruction [1]. Despite the substantial body of literature available to provide information to women considering this option, evidence suggests that women’s preparedness before this surgery is low [2]. Furthermore, making decisions about reconstruction before surgery may be stressful [3]. Studies suggest that better preparedness may improve patient expectations, support decision-making, and alleviate anxiety [4, 5].

Patients in our unit undergoing mastectomy and considering immediate reconstruction are shown post-operative photographs of other women who have previously undergone reconstructive surgery to aid understanding of potential outcomes and help them choose preferred reconstruction method. We currently achieve this via two-dimensional (2D) photographs, but three-dimensional (3D) images (digital models utilising computer rendered graphics) are an innovation we wished to include in pre-operative counselling sessions as a means of improving patient preparedness [6]. The aim of this study was to qualitatively explore the patient perspective after interacting with these 3D images.

## Methods

**Fig. 1:**
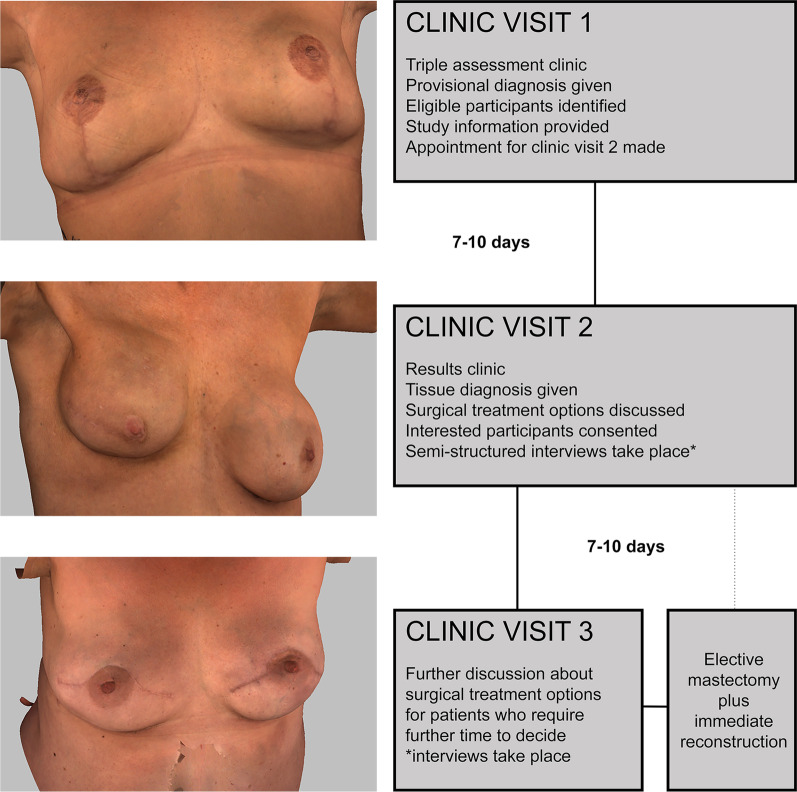
Examples of 3D images (left) and clinic recruitment/treatment schedule (right). *Interviews took place on visit 2 or 3 depending on participant preference. Triple assessment clinic = one stop clinic where clinical examination ± breast/axilla imaging ± biopsy for histology all take place during one visit

3D images were captured using 3dMD torso system, which produces a 190° high resolution graphic (Fig. 1) [7]. Fifty-two images of women who underwent mastectomy plus reconstruction were reviewed and seventeen selected to reflect variation in type of reconstruction surgery (implant or tissue flap), body habitus, and skin tone. Seven of these images deliberately showed sub-optimal aesthetic outcomes to give participants a balanced view of potential outcome.

Participants had the opportunity to engage with 3D images displayed using software on a laptop device during nurse specialist-led consultations. This included being able to observe, rotate, zoom in, and view reconstructed breast(s) from a variety of angles and aspects. Each woman was given the opportunity to view and interact with all seventeen 3D images with no upper limit of time specified for each interaction. Afterwards, women then spent between two to three minutes per image focusing on a smaller number of 3D images (i.e., no more than 3) selected by nurse specialists, which more closely matched patient skin tone, body habitus, and potential reconstruction method. Women diagnosed with breast cancer and undergoing mastectomy with immediate reconstruction were purposively sampled. Women not considering immediate reconstruction were excluded (Fig. 1).

Data was collected by AB via face-to-face semi-structured interviews conducted after participants interacted with the software (Table 1). A pilot interview was conducted between AB and a participant with no refinement. Interviews were audio-recorded and transcribed verbatim. No field notes were taken. Data was entered into NVivo for thematic analysis [8]. Themes were identified using an inductive reasoning approach. Two authors independently coded transcripts. Differences in coding were resolved via discussion. No new themes had emerged after analysis of transcripts and authors were therefore content that saturation was achieved [9]. There was no prior relationship between AB and participants.

**Table 1:** Semi-structured interview schedule questions 1–6

1	What is your overall opinion of the consultation you received?
2	Did this consultation trigger any particular emotions for you, and if so, what triggered those emotions?
3	Do you have thoughts or feelings about the inclusion of photographs of other women during the consultation?
4	Which type of photograph (2D or 3D) gave you a better idea about breast size and what makes you say this?
5	Which type of photograph (2D or 3D) gave you a better idea about breast symmetry (researcher allowed to explain meaning of this word if requested) and what makes you say this?
6	Was it useful to view images of women with similar skin colour, breast size, and type of surgery, and if so, why?

This report adheres to the 32-item COREQ checklist [10]. All methods were carried out in accordance with relevant guidelines and regulations under ethics approval and consent to participate. Study was given ethics approval by the Office of Research Ethics Committees Northern Ireland (Ref: 18/NI/0156) (Additional file 1).

## Results

Eight Caucasian women, labelled as participants (P) 1–8 below, aged 30–60 years participated (Table 2). A further one woman consented to participate but was excluded prior to viewing 3D images and interview because she had already made the decision to pursue delayed breast reconstruction surgery. Interviews lasted an average of 9 min (range 6–12 min). The major emergent theme from analysis was increased preparedness. Subthemes included expectation management, software interaction and enhanced realism. No participants expressed prohibitively negative emotions after viewing 3D images.

**Table 2:** Participant engagement with semi-structured interview questions; ✓ = question asked by interviewer and answered by participant, x = question either not asked by interviewer or not answered by participant

Participant	Question 1	Question 2	Question 3	Question 4	Question 5	Question 6
1	✓	✓	✓	✓	✓	✓
2	✓	✓	✓	x	✓	✓
3	✓	✓	✓	✓	✓	✓
4	✓	✓	✓	✓	x	✓
5	✓	✓	✓	x	✓	✓
6	✓	✓	✓	✓	✓	✓
7	✓	✓	✓	✓	✓	✓
8	✓	✓	✓	x	✓	✓

### Expectation management

‘you can get carried away in your head thinking *oh this is going to be gorgeous*… but it may not be like that, so I’d rather [have viewed 3D images] than come out the other end and say *but nobody told me*’ (P1). ‘I think that it gives you more of a perspective on what’s happening, you know, seeing what you’re going to go through’ (P4). A participant also mentioned that viewing 3D images left her ‘more confident, more informed going forward’ (P5).

### Software interaction

‘I liked the 3D images because you can manipulate it, especially when C said this is what you’re going to see when looking down [at your chest], I thought that was very useful’ (P6). ‘I think the 3D photographs were better because you could you know, move them around’ (P8). ‘I found that when I looked at the pictures that were printed out [2D], the implant to me seemed quite a viable option… It wasn’t until I seen the 3D scan and they were able to turn it around that I actually realised the shape isn’t as symmetrical as I thought’ (P1).

### Enhanced realism

‘They [3D images] were more realistic… because you sort of seen it from different angles, whereas in a [2D] photograph you’re just seeing it from one angle’ (P4). ‘I found the 3D images much better. I think they just looked more normal I suppose, where the straight on pictures probably didn’t look as normal’ (P2). A participant also remarked that ‘you know that’s somebody real in the 3D picture’ (P5).

## Discussion

Participants self-reported that viewing 3D images increased preparedness prior to surgery. Participants derived added value from their interactions with the software with regards to gaining a better appreciation of post-operative symmetry and a greater sense of realism. Reassuringly, participants reported no psychological distress after viewing 3D images.

In this report, participants mentioned feelings of increased preparedness and confidence before surgery after viewing 3D images. This is important, because over 40% of women undergoing mastectomy and reconstruction perceived their outcome to be worse than expected—particularly with regards to aesthetics [11]. Surgical teams already derive added value from utilising 3D images for aesthetic assessment after breast surgery [12]. Furthermore, an early phase randomised-controlled-trial suggested that viewing 3D images improved patient confidence going into surgery when compared with 2D photographs [13]. This report demonstrates that patients *also* value the ability to engage with 3D images to better appreciate potential post-operative outcomes with regards to appearance. We feel this will likely build confidence and improve decision-making.

Finally, we were interested to discover that several participants commented on 3D images being more realistic than 2D photographs. We hypothesise that viewing 3D images may increase preparedness prior to surgery by allowing patients to develop a more realistic understanding of what is actually achievable after breast reconstruction.

## Conclusion

Ongoing work is evaluating the use of decision-aids to enhance preparedness in women undergoing breast reconstruction [14, 15]. This report demonstrates that using 3D images during counselling sessions is an acceptable decision-aid, however, we acknowledge the small sample size, brevity of interviews, limited diversity amongst participants, and lack of post-operative follow up. In addition, the version of 3D technology utilised for this study did not yield a 360° image so women interested in assessing the aesthetic outcome from reconstruction procedures such as a latissimus dorsi flap were unable to fully appraise scars outside the breast region.

Our future work will aim to quantify benefit gained from viewing 3D images, using a version of technology capable of capturing 360° views, via an experimental study design whereby we assess both pre- and post-operative patient satisfaction scores. We also wish to qualitatively explore the opinions of nurse specialists delivering this intervention.

## Supplementary Information

**Additional file 1.** Semi-structured interview format.

**Additional file 2.** Participant information leaflet.

## Data Availability

Original interview transcripts available on reasonable request from the corresponding author.
