# Supplementary material for: Improving preparedness prior to reconstructive breast surgery via inclusion of 3D images during pre-operative counselling: a qualitative analysis
Source: BMC Womens Health. 2021 Aug 31;21:323. doi: 10.1186/s12905-021-01463-6 (PMC8408958; doi:10.1186/s12905-021-01463-6)
Supplement: Supplementary file 1 — Additional file 1. Semi-structured interview format. [file 12905_2021_1463_MOESM1_ESM.pdf]

Semi-structured interview guide for service user

Opening: Now that you have sat and discussed your surgery and have viewed both two-dimensional and three-dimensional images I was hoping to ask you a few questions about your thoughts and feelings about that consultation

1. What is your overall opinion of the consultation you received? (**general opening question**)

2. Did this consultation trigger any particular emotions for you, and if so, what triggered these emotions?

3. Do you have any thoughts or feelings about the inclusion of photographs of other women in the consultation?

4. Which type of photograph (2d or 3d) gave you a better idea about breast size and what makes you say this?

5. Which type of photograph (2d or 3d) gave you a better idea about breast symmetry (*researcher allowed to explain meaning of this word if requested*) and what makes you say this?

6. Was it useful to view images of women with similar skin colour, breast size, and type of surgery, and if so, why?
