# Supplementary material for: Improving preparedness prior to reconstructive breast surgery via inclusion of 3D images during pre-operative counselling: a qualitative analysis
Source: BMC Womens Health. 2021 Aug 31;21:323. doi: 10.1186/s12905-021-01463-6 (PMC8408958; doi:10.1186/s12905-021-01463-6)
Supplement: Supplementary file 2 — Additional file 2. Participant information leaflet. [file 12905_2021_1463_MOESM2_ESM.pdf]

Information leaflet for service user participants (version 2.2): 21/11/2018

Belfast Health and Social Care Trust
caring supporting improving together

QUEEN'S UNIVERSITY BELFAST

Project title: Service User and Service Provider Satisfaction with Three-Dimensional Breast Photographs as a Pre-Operative Decision-Making Aid

Background: Breast cancer is one of the most common cancers to affect women in Northern Ireland. For many women with breast cancer, surgical removal of the cancer is the main treatment method, although other treatments such as chemotherapy and radiotherapy may be used. Although there are different operations used to treat breast cancer, some women will require to have removal of the breast (a mastectomy). Many women choose to have some form of reconstruction after a mastectomy, and there are many different methods of carrying out breast reconstruction. Usually, the choice of method is made by a woman together with her breast surgeon. This can be a difficult decision for some women, and so this study is going to see whether 3d (three dimensional) images of breast surgery outcomes can be helpful to women when making decisions about their breast surgery and immediate reconstruction treatment.

Aim: the goal of this research is to determine if women, like yourself, find viewing images of other women who have undergone surgery and reconstruction similar to the one you may decide to undergo, helps in the decision-making process before your operation. We are also interested to find out what effect viewing of these images has on levels of worry and anxiety.

Who is involved? this study is being undertaken by breast surgeons working within the Belfast City Hospital and research scientists working within Queen's University Belfast. We will be interviewing women, like yourself, who have recently been diagnosed with breast cancer and are likely to undergo surgery. We will also be interviewing nurses and doctors involved in counselling women about different types of surgery for breast cancer.

About the study: you have been invited to take part in an interview that requires half an hour of your time to sit down to a discussion with a doctor. During this interview, your voice will be recorded using a tape recorder, so we can re-listen to and learn from what was said. Before this interview, you will talk with either a nurse or doctor about different types of surgery. Women normally get the chance to view photographs of other women who have been through similar surgeries. If you choose to take part in this study, you will get the chance to see an additional set of 3d images of women who have been through a similar surgery to the one you are likely to go through.

Do I have to take part and what are the benefits if I choose to? you do not need to take part, and not taking part will not influence your care. If you choose to take part, you will get the chance to contribute to research about how to better communicate with women who have been diagnosed with breast cancer.

Can I withdraw my participation? you are free to withdraw at any time of your choosing by emailing one of the project contacts listed below. Withdrawing your participation will not affect your care.

Are there any risks? we don't believe so, although if anything upsets or concerns you during the interview then a doctor will be available to talk with you.

Where will my data be stored? voice recordings will be stored anonymously within a secure password-controlled computer within the hospital and will be listened to again by researchers before being transcribed (copied down) in writing so we can perform further research on it. All voice recordings and written transcripts will only be accessed and used for the duration of this study. They will be deleted thereafter.

Who has reviewed this research? we are grateful to members of the Northern Ireland Cancer Research Consumer Forum who reviewed this study and suggested how to improve it. This group represents the interests of patients involved in research and we have happily taken their comments on board. This study has received a favourable opinion from Health and Social Care Research Ethics Committee A (HSC REC A).

What will happen to this research? we will aim to publish results from this research project in a peer-reviewed medical journal. If you wish to be kept updated on the publication of results or would like us to re-contact you for any future research studies you will be welcome to leave an email address for us to contact you with. We will only ever contact you to inform you about publication of results or if we are performing a follow up study on breast cancer surgery decision-making.

Who can I contact? please email Dr McCrorie (amccrorie02@qub.ac.uk) or Mr McIntosh (stuart.mcintosh@belfasttrust.hscni.net) with any enquiries. Mr McIntosh can be contacted via his secretary on 02895048337.

What if I have a complaint? If you wish to make a complaint or raise an issue with any aspect of this study then please contact either of the above researchers in the first instance. In the event that your complaint remains unresolved, please email complaints@belfasttrust.hscni.net or telephone 02895048000.

Privacy notice: Queen's University Belfast is the sponsor for this study based in the United Kingdom. We will be using information from you in order to undertake this study and will act as the data controller for this study. This means that we are responsible for looking after your information and using it properly. Queen's University Belfast will keep identifiable information about you for five years after this study has finished. Your rights to access, change or move your information are limited, as we need to manage your information in specific ways in order for the research to be reliable and accurate. If you withdraw from the study, we will keep the information about you that we have already obtained. To safeguard your rights, we will use the minimum personally-identifiable information possible. You can find out more about how we use your information by contacting **info.compliance@qub.ac.uk.**

As a university we use personally-identifiable information to conduct research to improve health, care and services. As a publicly-funded organisation, we have to ensure that it is in the public interest when we use personally-identifiable information from people who have agreed to take part in research. This means that when you agree to take part in a research study, we will use your data in the ways needed to conduct and analyse the research study. Your rights to access, change or move your information are limited, as we need to manage your information in specific ways in order for the research to be reliable and accurate. If you withdraw from the study, we will keep the information about you that we have already obtained. To safeguard your rights, we will use the minimum personally-identifiable information possible. Health and care research should serve the public interest, which means that we have to demonstrate that our research serves the interests of society as a whole. We do this by following the **UK Policy Framework for Health and Social Care Research.** If you wish to raise a complaint on how we have handled your personal data, you can contact our Data Protection Officer who will investigate the matter. If you are not satisfied with our response or believe we are processing your personal data in a way that is not lawful you can complain to the Information Commissioner's Office (ICO).

Our Data Protection Officer is **Derek Weir** and you can contact them at **info.compliance@qub.ac.uk**. He is based within the registrar's office, Lanyon South, Queen's University Belfast BT7 1NN.

Belfast trust will collect information from you for this research study in accordance with our instructions. Belfast trust will keep your name and contact details confidential and will not pass this information to any outside organisation. Belfast trust will use this information as needed, to contact you about the research study, and make sure that relevant information about the study is recorded for your care, and to oversee the quality of the study. Certain individuals from Queen's University Belfast may look at your medical and research records to check the accuracy of the research study. Queen's University Belfast will only receive information without any identifying information. The people who analyse the information will not be able to identify you and will not be able to find out your name, H&C number or contact details. Belfast trust will keep identifiable information about you from this study for five years after this study has finished.
